# Revisiting the Ventral Medial Nucleus of the Hypothalamus: The Roles of SF-1 Neurons in Energy Homeostasis

**DOI:** 10.3389/fnins.2013.00071

**Published:** 2013-05-07

**Authors:** Yun-Hee Choi, Teppei Fujikawa, Jiwon Lee, Anne Reuter, Ki Woo Kim

**Affiliations:** ^1^Division of Hypothalamic Research, Department of Internal Medicine, University of Texas Southwestern Medical CenterDallas, TX, USA; ^2^Department of Pharmacology, University of Texas Southwestern Medical CenterDallas, TX, USA; ^3^Department of Research Administration, University of Texas Southwestern Medical CenterDallas, TX, USA; ^4^Department of Pharmacology, Institute of Lifestyle Medicine and Nuclear Receptor Research Consortium, Wonju College of Medicine, Yonsei UniversityWonju, Korea

**Keywords:** arcuate nucleus of the hypothalamus, energy homeostasis, glucose homeostasis, knockout, obesity, steroidogenic factor-1, ventral medial hypothalamic nucleus

## Abstract

Obesity, diabetes, and other metabolic complications are growing concerns for public health and could lead to detrimental life-threatening conditions. Neurons whose activities are required for energy and glucose homeostasis are found in a number of hypothalamic nuclei. In the early twentieth century, the ventral medial nucleus of the hypothalamus (VMH) was the first site reported to play a prominent role in the regulation of energy homeostasis through control of food intake and energy expenditure. Recent studies using sophisticated genetic tools have further highlighted the importance of the VMH and have extended our understanding of the physiological role of the nucleus in regulation of energy homeostasis. These genetic studies were preceded by the identification of steroidogenic factor-1 (SF-1) as a marker of the VMH. This review focuses on the emerging homeostatic roles of the SF-1 neurons in the VMH discovered through the use of genetic models, particularly highlighting the control of energy, and glucose homeostasis.

## Introduction

Metabolic complications such as obesity, diabetes, and insulin resistance are growing public health concerns, and their incidences are continuously increasing. The National Health and Nutrition Examination Survey (NHANES) found that, from 2009–2010, the prevalence of overweight (BMI ≥ 25) for adults in the United States was 69.2% and the obesity rate (BMI ≥ 30) was 35.9% (Flegal et al., 2012). Imbalances in whole body glucose and insulin homeostasis are also closely linked to the trend of increased obesity. Therefore, understanding the molecular and cellular mechanisms underlying energy balance, glucose, and insulin homeostasis is critical for developing new strategies for the prevention and treatment of metabolic syndromes including obesity and diabetes. The hypothalamus, especially the mediobasal hypothalamus, has been considered a site for whole body homeostatic regulation including body weight and energy metabolism. Although classical approaches, such as electronic/chemical lesioning or microinjection of a compound into a particular site have greatly expanded our knowledge of the physiological roles of mediobasal hypothalamic sites, they have caveats which limit our interpretations from those studies. For instance, electronic/chemical lesioning of the VMH likely damages the surrounding regions or the descending fibers passing through the VMH (Devenport and Balagura, 1971; Gold, 1973). In addition, the microinjection of any compounds into the hypothalamic site is prone to off-target effects (e.g., leakage of compounds outside of targeted site), which may compromise the analysis. The development of new genetic technologies including gene manipulation techniques at the level of specific neuronal populations can allow us to overcome these caveats and provides an advanced opportunity to investigate the neuronal circuitry underlying the mechanism of energy balance in a specific hypothalamic nucleus. We discuss, in this review, the significance of the VMH among many known hypothalamic nuclei in the regulation of energy and glucose homeostasis, which have been revealed by genetic approaches. More specifically, we will focus on steroidogenic factor-1 (SF-1) neurons, a representative VMH neuronal population, and its metabolic roles.

## The VMH: A Historic Landmark of Central Regulation of Metabolism

**Figure 1:**
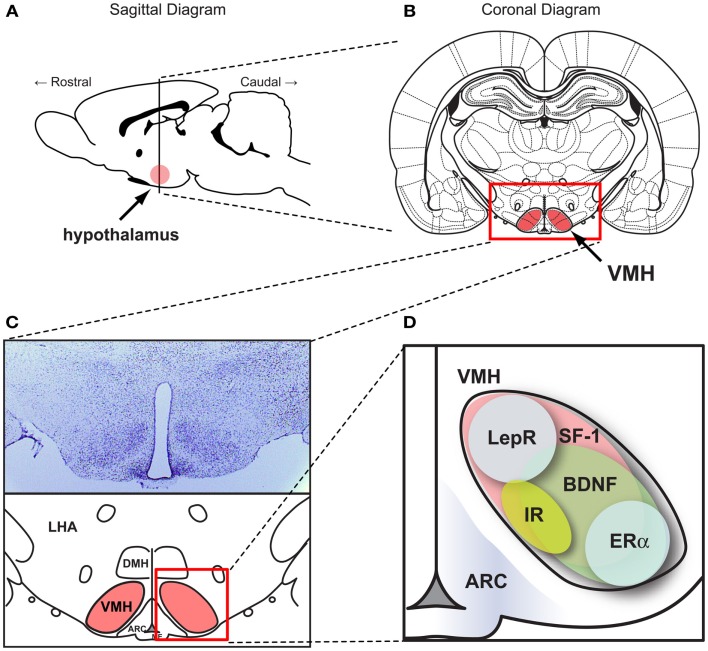
**SF-1 neurons in the ventromedial hypothalamic nucleus (VMH)**. Figures show **(A)** the location of the hypothalamus in the CNS in a sagittal section and **(B)** the location of VMH in a coronal section. **(C)** (Upper) Nissle stained coronal section of the hypothalamus and (lower) schematic diagram of the hypothalamus within the coronal section. **(D)** Schematic diagram showing the pattern of genes expressed in the VMH. ARC, the arcuate nucleus. DMH, dorsomedial hypothalamic nucleus. LHA, lateral hypothalamic area. VMH, ventral medial nucleus of the hypothalamus. BDNF, brain-derived neurotrophic factor. ERα, estrogen receptor alpha. IR, insulin receptors. LepR, leptin receptors. SF-1, steroidogenic factor-1. Schematic diagrams were generated based on data from McClellan et al. (2006) and Yi et al. (2011).

The hypothalamus, comprised of several distinct nuclei, is a critical center for homeostatic regulation in the central nervous system. Among several hypothalamic nuclei, the VMH was the first site which was recognized as a site for body weight regulation and energy homeostasis (Hetherington, 1941). Since then, the VMH has remained site of interest for body weight regulation and glucose homeostasis. (Hetherington, 1941; Rothwell and Stock, 1979; Minokoshi et al., 1986; Amir, 1990; Dhillon et al., 2006; King, 2006; Bingham et al., 2008; Klockener et al., 2011). It has been convincingly shown that VMH lesions directly impact body weight and food intake mainly through the regulation of the autonomic nervous system (King, 2006). The VMH, a nucleus in the mediobasal hypothalamus, is a bilateral cell group with an elliptical shape located above the median eminence (Figure 1). The cytoarchitecture of the VMH can be distinctively defined by various histological methods including Nissl staining because the VMH is surrounded by a cell-poor/fiber (dendrite)-rich zone (Figure 1). The VMH cytoarchitecture is detectable as early as embryonic day 15 (E15) (McClellan et al., 2006).

The molecular and cellular mechanisms by which the CNS including the VMH regulates body weight, food intake, energy, and glucose homeostasis had been relatively unclear until leptin and its cognate receptors (LEPRs) were identified (Zhang et al., 1994; Tartaglia et al., 1995). Although many other hormones such as insulin have been thought to affect the CNS to regulate energy and glucose homeostasis, it is safe to say that the identification of leptin in white adipose tissues and discovery that LEPRs are mainly expressed in the CNS opened the door for a new era of study on central regulation of homeostasis. Several studies have pointed to the arcuate nucleus (ARC) of the hypothalamus, specifically the proopiomelanocortin (POMC)/agouti-related peptide and neuropeptide Y (AgRP/NPY)-expressing neurons, as the primary target of leptin and insulin action (Williams et al., 2010; Gautron et al., 2011; Vianna and Coppari, 2011). Besides juxtaposed POMC and AgRP neurons in the ARC, the VMH expresses LEPRs and is a targeted site of leptin and insulin for the regulation of energy and glucose homeostasis (Elmquist et al., 1998; Scott et al., 2009). For example, direct application of leptin into the VMH preferentially increased glucose uptake in skeletal muscle, heart, and brown adipose tissue (BAT), and this increased glucose uptake was impaired when the sympathetic nervous system (SNS) was denervated, suggesting that leptin signaling in the VMH plays crucial roles in mediation of sympathetic tone from the VMH to peripheral tissues (Hetherington and Ranson, 1940; Kamohara et al., 1997; Haque et al., 1999; Minokoshi et al., 1999; Toda et al., 2009; Kim et al., 2012). Although classic studies had extended our knowledge on the VMH, recent genetic studies have obliged us to reconsider and re-highlight the physiological roles of the VMH in the regulation of energy and glucose homeostasis.

## SF-1 in the CNS: A Marker of the VMH

The VMH is comprised of various cell types with heterogeneous gene expression patterns. Many of the genes highly expressed in the VMH have been identified and their functions have been studied (McClellan et al., 2006). Among them, cannabinoid receptor 1 (CB1), pituitary adenylate cyclase activating polypeptide (PACAP), brain-derived neurotrophic factor (BDNF), and cerebellin 1 (Cbln1) which are broadly expressed throughout the VMH (Xu et al., 2003; Segal et al., 2005; Kim et al., 2008, 2009; Liao et al., 2012). Other genes such as estrogen receptor alpha (ERα) and progesterone receptor (PR) possess a limited expression pattern restricted to the ventro-lateral portion of the VMH (Figure 1) (Musatov et al., 2006, 2007; Kim et al., 2010). One of our recent studies using laser-capture microdissection revealed VMH-enriched genes compared to the other hypothalamic regions (Segal et al., 2005). A similar study by the Ingraham laboratory identified approximately 200 genes highly enriched in neonatal (postnatal day 0) mouse VMH tissue, revealing distinct regional patterning in the newly formed VMH (Kurrasch et al., 2007). Of these identified genes, SF-1 (NR5A1) is a transcription factor that is required not only for terminal differentiation of the nucleus, but also for several VMH-mediated physiological processes including energy homeostasis (Majdic et al., 2002; Segal et al., 2005; Kurrasch et al., 2007). Intriguingly, although various genes are expressed in the VMH, SF-1 is the only gene which is specifically and exclusively expressed in the VMH. Indeed, development of SF-1 neuron-specific Cre lines in which Cre activity is limited to the VMH accelerated the functional understanding of genes expressed in the VMH (Bingham et al., 2006; Dhillon et al., 2006). A large body of literature now suggests that SF-1 neurons in the VMH play an important role in the regulation of energy and glucose metabolism.

## SF-1 Neurons Regulate Energy Homeostasis: Their Role as Thermogenic Neurons

### SF-1 in the VMH

As described above, SF-1-expressing neurons (SF-1 neurons), among the vast heterogeneous neuronal populations in the VMH, have recently emerged as a representative neuronal population involved in many aspects of metabolic regulation. Identification of the transcription factor SF-1 as an important metabolic regulator was established by the Parker group while studying germline SF-1 KO mice which exhibit neonatal lethality due to adrenal insufficiency (Majdic et al., 2002). To circumvent the death of SF-1 KO mice, they transplanted WT adrenal glands into the SF-1 KO animals. To their surprise, the transplanted SF-1 KO animals exhibited massive obesity compared to WT counterparts, thus suggesting that the expression of SF-1 in the VMH plays critical suppressive roles against body weight gain. However, since the VMH of germline SF-1 KO mice had disrupted cytoarchitecture, the obesity phenotype in the transplanted animals could be a consequence of impaired VMH formation rather than SF-1 absence in the nucleus (Majdic et al., 2002; Davis et al., 2004; Zhao et al., 2008). Therefore, we generated an alternative mouse model named postnatal VMH-specific SF-1 KO in which SF-1 is specifically deleted after completion of VMH development using CamKII-Cre, a postnatally expressing Cre line, to delineate the direct metabolic roles of SF-1 without the confounding developmental side effects (Kim et al., 2011). The studies using the postnatal VMH-specific SF-1 KO animals revealed that SF-1 is indeed required for normal energy homeostasis by modulating energy expenditure specially in high-fat diet condition (Kim et al., 2011).

### Manipulation of genes in SF1 neurons

Two independent groups generated SF-1-Cre transgenic lines in which the expression of Cre recombinase is restricted to SF-1 cells of the VMH, significantly advancing our understanding of VMH-mediated metabolic regulation (Bingham et al., 2006, 2008; Dhillon et al., 2006). These transgenic mice induce Cre-recombination in exclusively in the VMH. These lines were used to target the deletion of the leptin receptor and the insulin receptor in the VMH, providing direct physiological insights on their roles in the nucleus (Dhillon et al., 2006; Bingham et al., 2008; Klockener et al., 2011). Deletion of the leptin receptor in SF-1 neurons of the VMH (*Sf1-Cre, Lepr^flox/flox^* mice) resulted in similar effects on body weight regulation observed when the leptin receptor is removed from POMC neurons (*Pomc*-Cre, *Lepr^flox/flox^* mice) of the ARC (Balthasar et al., 2004; Dhillon et al., 2006). For example, both KO models significantly gained weight during 5–9 weeks of age mainly due to decreased energy expenditure but the weight gain were bunted afterward, implicating possible developmental compensatory mechanisms in adulthood in KO animals (Balthasar et al., 2004; Dhillon et al., 2006). Thus, additional studies using temporally manageable Cre lines would be helpful to explore direct metabolic roles of leptin receptor signaling in the VMH without the confounding effects of developmental compensation.

**Figure 2:**
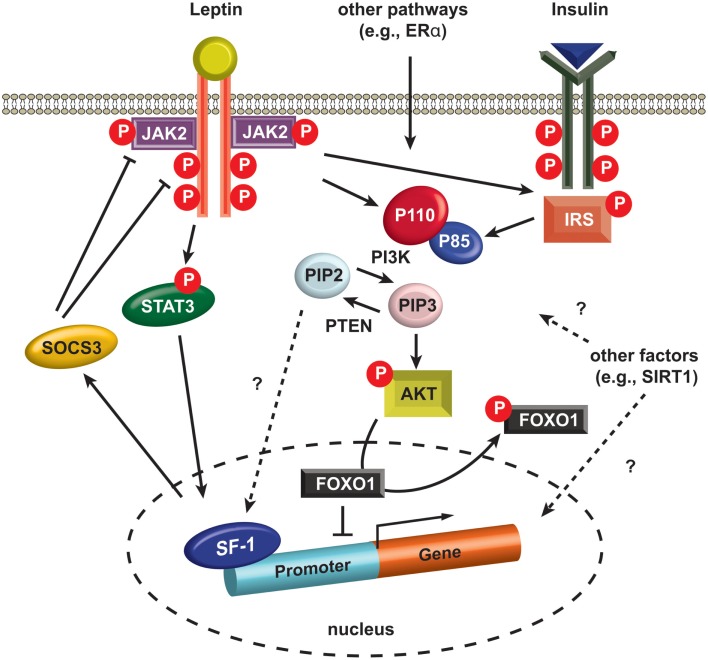
**Intracellular signaling in SF-1 neurons**. Leptin binding to LEPRs induces auto-phosphorylation of LEPRs and phosphorylation of JAK2 (pJAK2), and subsequently these phosphorylated sites and pJAK2 induce phosphorylation of STAT3 (pSTAT3). pSTAT3 is translocated into the nucleus and alters the expression of several genes including SOCS3. SOCS3 negatively regulates JAK2 and other phosphorylated sites in LEPRs. Insulin binding to insulin receptors activates IRS and it activates PI3K. PI3K phosphorylates PIP2 and produces PIP3. PTEN dephosphorylates PIP3, thus generating PIP2. A recent study indicates PIP2 directly binds SF-1 in the nucleus (Blind et al., 2012). PIP3 phosphorylates AKT (pAKT) and pAKT induces phosphorylation of FOXO1 (pFOXO1) in the nucleus. pFOXO1 is translocated to the cytosol. FOXO1 directly binds SF-1 and inhibit SF-1 activity. AKT, serine/threonine-specific protein kinase. FOXO1, Forkhead box protein O1. JAK2, janus kinase 2. IRS, insulin receptor substrate. STAT3, signal transducer and activator of transcription. PIP2, phosphatidylinositol (4,5)-bisphosphate. PIP3, phosphatidylinositol (3,4,5)-triphosphate, PTEN, phosphatase and tensin homolog. P110, p110 catalytic subunit. P85, p85 regulatory subunit. SIRT1, sirtuin 1 or silent mating type information regulation 2. SF-1, steroidogenic factor-1. SOCS3, suppressor of cytokine signaling-3.

The first direct evidence for insulin receptor signaling in the VMH was revealed by the generation of SF-1 neuron-specific insulin receptor knockout mice (SF-1^ΔIR^ mice) (Klockener et al., 2011). The SF-1^ΔIR^ mice exhibited improved glucose metabolism and resistance to high-fat diet, and, interestingly, increased cellular activity of POMC neurons. These observations suggest that the blunted insulin responses in SF-1 neurons in response to high-fat diet conditions increase excitatory neuronal projections to the POMC neurons resulting in protection from diet-induced weight gain. Notably, electrophysiological techniques used by the investigators revealed that the leptin- and insulin-responsive neurons are segregated in the SF-1 neurons as they are in POMC neurons (Williams et al., 2010; Klockener et al., 2011). In addition, reduced activity of *pik3ca* (p110α), a catalytic subunit of PI3K, in SF-1 cells rendered blunted autonomic responses to high calorie food and reduced leptin action without alteration in glucose and insulin homeostasis (Xu et al., 2010). Interestingly, specific deletion of a transcription factor, FOXO1, a downstream effector of PI3K, in SF-1 neurons results in profound effects on energy homeostasis together with alterations in glucose and insulin metabolism (Kim et al., 2012). Specifically, ablation of FOXO1 in SF-1 neurons exhibited lean phenotypes due to increased energy expenditure and enhanced insulin sensitivity by increment of insulin-stimulated glucose disposal in cardiac and skeletal muscle. These results indicate the presence of isoform-specific roles of PI3K in glucose and insulin homeostasis in the SF-1 neurons of the VMH (Figure 2).

Not surprisingly, investigators also used SF-1-Cre transgenic mice to examine the metabolic roles of several other genes thought to be associated with metabolic regulation. Enhanced leptin receptor signaling by removing suppressor of cytokine signaling-3 (SOCS3), a negative regulator of leptin action (Figure 2), from SF-1 neurons results in minimal effects on body weight but shows increased insulin sensitivity (Zhang et al., 2008). Furthermore, both inhibition and activation studies of SIRT1 in SF-1 neurons, demonstrated the protective roles of SIRT1 against diet-induced metabolic imbalance (Ramadori et al., 2011). More recently, we ablated estrogen signaling specifically in the ventro-lateral portion of the VMH using the SF-1-Cre transgenic mice revealing that estrogen signaling is required for normal energy expenditure and abdominal fat homeostasis (Xu et al., 2011).

Interestingly, at this point the aforementioned genetic studies on SF-1 neurons have argued the idea that the VMH is the center of satiety, as most of the studies did not report changes in food intake behavior (Table 1). Of note, SF-1 neurons are representative population of the VMH, yet SF-1 is not expressed in the entire nucleus. Thus, non-SF-1 neurons in the VMH may play a role in the regulation of food intake behavior. Indeed, deletion of long form 3UTR′ BDNF in the VMH leads to hyperphagia and obesity in mice (Liao et al., 2012). Moreover, deletion of ERα in the entire VMH leads to hyperphagia and more profound obesity (Musatov et al., 2007) than that seen when ERα is deleted only in SF-1 neurons (Xu et al., 2011). Notably, both BDNF and ERα are abundantly expressed in the ventro-lateral area of the VMH (Xu et al., 2003; Musatov et al., 2007), where SF-1 is not expressed in the adult (Cheung et al., 2013). Thus, it seems that topographically and genetically distinct neurons from SF-1 neurons may regulate food intake behavior. Contrarily, studies on SF-1 neurons support the idea that the VMH is the regulatory center of energy expenditure. A common characteristic among mice bearing genetically engineered gene deletions in SF-1 neurons is a defect in diet-induced thermogenesis upon high-fat diet feeding (Table 1). These mice do not exhibit metabolic dysfunction upon normal chow-diet; however, exposure to high-fat diet induces a defective ability to adapt to the thermogenic environment (we defined “HFD-induced phenotype.” Table 1). It is well known that high-fat diet feeding increases energy expenditure, thus heat production, which probably helps to avoid excessive body weight gain. Although none of the studies described above investigated whether SF-1 neurons regulate thermogenesis/energy expenditure under other thermogenic environments such as cold exposure or prolonged exercise, SF-1 neurons may play roles in the regulation of thermogenesis/energy expenditure under those thermogenic environments as well. Collectively, although SF-1 neurons do not represent all neurons within the VMH, the genetic deletion or overexpression studies completed to date establish that SF-1 neurons in the VMH are essential for normal energy homeostasis, particular in regulation of thermogenesis/energy expenditure.

**Table 1:** **Gene manipulation specifically in SF-1 neurons affects energy and glucose homeostasis**.

Molecule	Diet	Bodyweight	Food intake	Energy expenditure	Adiposity	Glycemia	Insulin sensitivity	leptin sensitivity	Reference
SF1 (prenatal)	SD	△ (female in old ages)	-	-	?	?	?	?	Kim et al. (2011)
	HFD	△	-	?	△	?	?	?	
SF1 (postnatal)	SD	-	-	-	-	△ (fed and fast in old ages)	▼	▼	
	HFD	△	-	▼	△	△ (fed and fast)	?	?	
LepR	SD	△	-	-	△	-	?	?	Dhillon et al. (2006)
	HFD	△	△	▼	△	-	?	?	
IR	SD	-	-	-	-	-	?	?	Klockener et al. (2011)
	HFD	▼	▼	-	▼	-	△	△	
PTEN	SD	△	△	?	?	?	?	?	
	HFD	-	-	?	?	?	?	?	
p110α	SD	-	-	-	-	-	-	▼	Xu et al. (2010)
	HFD	△	-	▼	△	?	?	?	
FOXO1	SD	▼	-	△	▼	▼ (fed and fast)	△	△	Kim et al. (2012)
	HFD	▼	-	△	▼	?	?	?	
SOCS3	SD	-	▼	▼	?	▼ (fed and fast)	△	△	Zhang et al. (2008)
	HFD	-	▼	▼	?	▼ (fed)	△	△	
SIRT1 (KO)	SD	-	-	-	-	-	?	?	Ramadori et al. (2011)
	HFD	△	-	▼	△	△ (fed and fast)	▼	?	
SIRT1(OE)	SD	-	-	-	-	-	-	-	
	HFD	▼	-	△	▼	-	△	?	
ERα	SD	-	-	-	-	-	▼ (female)	?	Xu et al. (2011)
	HFD	△ (female)	-	▼ (female)	△ (female)	-	?	?	
VGLUT2	SD	-	?	?	?	▼ (fast)	?	?	Tong et al. (2007)
	HFD	△	△	-	△	?	?	?	

*All genes except for SIRT1 were deleted specifically in SF-1 neurons. -, unchanged. △, increased. ▼, decreased. ?, not measured. SD, standard diet. HFD, high-fat diet. KO, knockout. OE, overexpression. SF-1, steroidogenic factor-1. LepR, leptin receptors. IR, insulin receptors. PTEN, phosphatase and tensin homolog. P110 α, p110 catalytic subunit alpha. FOXO1, forkhead box protein O1. SOCS3, suppressor of cytokine signaling-3. SIRT1, Sirtuin 1 or silent mating type information regulation 2, homolog 1. ERα, estrogen receptor alpha. VGLUT2, vesicular glutamate transporter 2*.

### Comparison to other hypothalamic neurons

Some physiological roles (e.g., regulation of food intake and bodyweight) among POMC, AgRP, SF-1, and other hypothalamic neurons likely overlap because those neurons directly and indirectly communicate with each other within the hypothalamus. Indeed, redundant roles should exist to maintain energy homeostasis which is fundamental for life. Therefore, it is difficult to explicitly define the distinct roles that different neurons have in the regulation of energy homeostasis. For instance, deletion of LEPRs either in POMC or SF-1 neurons leads to the same degree of body weight gain in chow-diet feeding. Nonetheless, it must be noted that in most cases, mice with a manipulated gene in POMC or AgRP neurons exhibit metabolic abnormalities under even chow-diet feeding conditions (Vianna and Coppari, 2011). Meanwhile, as see in Table 1, HFD-induced phenotypes are prominently observed in mice with a manipulated gene in SF-1 neurons. Thus one distinct physiological role of SF-1 neurons compared to other hypothalamic neurons is that they may regulate energy homeostasis to adapt to thermogenic, at least obesogenic, environments. Since a large number of studies indicate that induction of thermogenesis may be useful to treat obesity and eventually obesity-related disease, SF-1 neurons are a potential target for development of anti-obesity drugs.

## SF-1 Neurons Regulate Glucose Homeostasis: Sensing and Regulating Glucose

Besides the important role of SF-1 neurons in the regulation of energy homeostasis, genetic studies have also illuminated that SF-1 neurons are critical components of mechanism underpinning glucose homeostasis. While it has been suggested that the hypothalamus and the VMH have a role in the control of glucose levels and counterregulation, conclusive evidence was lacking (Himsworth, 1970; Niijima et al., 1988). The first clear evidence indicating that the VMH is a key glucose-sensing region was established about two decades ago (Borg et al., 1994). Borg et al. (1994) reported that conscious rats with a chemical lesion in the VMH exhibited impaired glucagon, epinephrine, and norepinephrine responses against hypoglycemia. In addition, inducing glucopenia around the VMH using 2-deoxyglucose (a non-metabolizable glucose analog), resulted in an immediate increase in plasma glucose in association with a marked elevation of glucagon, epinephrine, and norepinephrine, suggesting that neurons in the VMH play a critical role in triggering the release of counterregulatory hormones important for defending against hypoglycemia (Borg et al., 1995). They convincingly established the VMH as a key brain site for hypoglycemic counterregulation by producing hypoglycemia only in peripheral tissues (Borg et al., 1997). Combined with these physiological studies, several electrophysiological studies further demonstrated that the VMH contains neurons that directly respond to glucose (Song and Routh, 2006; Cotero and Routh, 2009).

Tong et al. (2007) recently demonstrated a critical role for the vesicular glutamate transporter (VGLUT2) in counterregulatory action against insulin-induced hypoglycemia specifically in SF-1 neurons of the VMH. In this study, they generated mice lacking VGLUT2 selectively in SF-1 neurons to determine the metabolic roles of the fast-acting neurotransmitter glutamate. They found that the KO mice (*Sf1-Cre*;*Vglut2^flox/flox^*) were slightly obese compared to their controls, especially when challenged with a high-fat diet. Interestingly, the *Sf1-Cre*;*Vglut2^flox/flox^* mice exhibited impaired glucose homeostasis in the fasted condition together with blunted counterregulatory responses to insulin-induced hypoglycemia. Furthermore, when they induced hypoglycemia using a hypoglycemic clamp, the *Sf1-Cre*;*Vglut2^flox/flox^* mice displayed significantly blunted responses of counterregulatory hormones including glucagon and epinephrine. This genetic approach establishes that SF-1 neurons of the VMH are a critical subset of neurons playing important roles in counterregulation against hypoglycemia in the brain.

In addition, accumulating evidence has pointed to leptin, insulin, and their downstream effectors as regulators of glucose homeostasis in the SF-1 neurons. For example, the restoration of leptin receptors within the mediobasal hypothalamus of leptin receptor-mutated Koletsky rat using virus-mediated gene delivery significantly increased glucose and insulin sensitivity through SNS activity. This improved sensitivity was blunted by application of a PI3K inhibitor, suggesting that the leptin-PI3K-FoxO1 pathway is a potential signaling pathway for glycemic homeostasis in SF-1 neurons (Keen-Rhinehart et al., 2005; Morton et al., 2005; Morton and Schwartz, 2011; Kim et al., 2012). Together with the leptin-PI3K-FoxO1 pathways, a genetic KO study suggested that the leptin-phosphorylated signal transducer and activator of transcription 3 (pSTAT3) pathway is also associated with glycemic control in SF-1 neurons of the VMH (Zhang et al., 2008). A recent report indicated that microinjection of orexin into the VMH enhances glucose uptake in the skeletal muscle (Shiuchi et al., 2009). In line with this report, deletion of SIRT1 in SF1 neurons attenuates orexin induced activation of the SF1 neurons, and this underlies decreased insulin sensitivity in the skeletal muscle of SF1-neuron-specific SIRT1 KO mice (Ramadori et al., 2011). Moreover, a recent study identified that the transcriptional programs of SF-1 are required for the normal glucose homeostasis in rodents, especially in relatively old age groups (Kim et al., 2012). Therefore, it is entirely possible that transcriptional networks regulated by SF-1, pSTAT3, and FoxO1 that are responsible for the modulation of neuronal activities from or in the VMH might be crucial for glucose and energy homeostasis. Future studies dissecting these complicated networks would be helpful not only to understand the glycemic homeostasis regulated by the brain but also to facilitate drug development to possibly treat diabetes.

## Perspectives: Novel Genetic Tools for Understanding SF-1 Neurons

Studies using genetically engineered mice have provide more specific and precise insights by which the hypothalamic neurocircuitry regulates energy homeostasis than classical approaches. However, a major critique of traditional knockout mouse models is that genetic modifications that occur prenatally may be affected by developmental plasticity/compensation, especially in the CNS. This issue has been highlighted by work from Palmiter and colleagues who have found disparate phenotypes in mice with prenatal and postnatal ablation of AgRP neurons (Luquet et al., 2005, 2007; Wu et al., 2008, 2009). Briefly, global knockout of AgRP neurons does not cause any abnormalities of body weight, food intake behavior and adiposity (Luquet et al., 2007). Deletion of AgRP neurons early in development by diphtheria toxin-inducible system reveals that prenatal deletion of AgRP leads to modest changes in body weight; however, deletion of AgRP neurons in adulthood results in severe hypophagia, decreased body weight and eventually causes death (Luquet et al., 2005). Moreover, it has been shown that prenatal mice express POMC in multiple hypothalamic lineages, including cells that do not express POMC in adult mice (Padilla et al., 2010). This developmental issue is of particular concern in future studies on SF-1 neurons as a recent report shows that the expression pattern of SF-1 in the CNS differs between prenatal and adult mice (Cheung et al., 2013). In this regard, for instance, generating an inducible SF-1-Cre mouse must be a top priority to avoid a developmental caveat seen in a conventional SF-1-Cre mouse.

Recent advanced genetic technologies, optogenetic and pharmacogenetic tools, have been employed to probe the function of neuronal activities and the neuronal circuits underpinning food intake and reward behavior (Domingos et al., 2011; Krashes et al., 2011; Kong et al., 2012). Detailed methodology of optogenics and pharmacogenetics are described elsewhere, but briefly these techniques can allow us to temporally and precisely manipulate particular neuronal activities either *in vitro* or *in vivo* in adult rodents. Optogenetic approaches take advantage of genetically engineered photochemical receptors, for instance, Channel-rhodopsin-2 (ChR2), which is isolated from *Chlamydomonas reinhardtii*^1^. Particular wavelengths of light (e.g., 480 nm for ChR2) can stimulate optogenetical receptors and lead to depolarization (activation) or hyperpolarization (inhibition) of the membrane potential of neuronal cells. Pharmacogenetic tools such as Designer Receptor Exclusively Activated by Designer Drugs (DREADD) which is a genetically modified G-coupled protein receptor, has been developed by Roth and colleagues^2^. Neurons expressing DREADD can be specifically activated or inhibited by an administration of clozapine-N-oxide (CNO). Compared to optogenetic tools, pharmacogenetic tools can allow us to modulate neuronal activity in specific neurons for hours by just a single administration of CNO (Krashes et al., 2011). These advanced techniques have great advantages over conventional Cre/loxP technology. First, we can examine physiological outcomes of “direct” manipulation of the excitability specific of neurons *in vivo* in adult rodents. Second, using these two tools combined with other pharmacological and neuronal tracing techniques we can now map the neuronal circuit along with physiological outcomes (Atasoy et al., 2012). Yet these advanced technologies have not been applied to discerning the physiological role of SF-1 neurons and the neuronal circuit of SF-1 neurons. Specifically, these technologies may answer many questions: what are the metabolic outcomes (e.g., alterations in energy expenditure and glucose levels in the blood) of alterations in the membrane potential of SF-1 neurons?; and what neurons connected to SF1 neurons are important to regulate energy and glucose homeostasis? Moreover, those technologies may unravel the intra-VMH connectome, which is completely unknown. Those answers undoubtedly will be instrumental to design new therapies for obesity and obesity-related diseases.

## Summary

It is now established that the hypothalamus is a major site for the regulation of metabolic homeostasis in the brain. Disruption of this homeostasis leads to obesity, diabetes, and other metabolic complications. Although we have witnessed marked advances in the field of central regulation of energy and glucose homeostasis during the past century, the hypothalamic circuitry is far more complicated and needs to be further investigated. As described above, because neurons within the hypothalamus communicate with each other and have redundant physiological roles, it is virtually impossible to clearly determine the division of roles in energy regulation and glucose homeostasis among hypothalamic neurons. Nonetheless, it is worthwhile to emphasize that SF-1 neurons play critical roles in the regulation of energy homeostasis to adapt to obesogenic and likely other thermogenic environments, than other hypothalamic neurons. Moreover, genetic studies indicate that SF-1 neurons may mediate the effect of various hormones including leptin and orexin on glucose metabolism. However, several pieces of knowledge for understanding the mechanism by which SF-1 neurons regulate energy homeostasis, particularly in thermogenesis, and glucose homeostasis are missing. Novel genetic tools, which are described above and will be developed in the future, will unravel that mechanism and ultimately may lead to the design of new anti-obesity and -diabetes drugs.

## Conflict of Interest Statement

The authors declare that the research was conducted in the absence of any commercial or financial relationships that could be construed as a potential conflict of Interest.

## Footnotes

^1^http://www.stanford.edu/group/dlab/optogenetics/

^2^http://pdspit3.mml.unc.edu/projects/dreadd/wiki/WikiStart
